# Emergence of KPC-2 and NDM-13-coproducing carbapenem-resistant hypervirulent *Klebsiella pneumoniae* with high-risk sequence type ST11

**DOI:** 10.3389/fmicb.2025.1683102

**Published:** 2025-12-17

**Authors:** Jia Tao, Liru Wang, Na Yang, Hui Fu, Xinxin Hu, Tingyu Lai, Jianning Jin, Gang Li

**Affiliations:** 1 Center of Medical Laboratory, General Hospital of Ningxia Medical University, Yinchuan, China; 2 Ningxia Key Laboratory of Clinical and Pathogenic Microbiology, General Hospital of Ningxia Medical University, Yinchuan, China; 3 Traditional Chinese Medicine Department, General Hospital of Ningxia Medical University, Yinchuan, China; 4 The First Clinical Medical College, Ningxia Medical University, Yinchuan, China; 5 School of Laboratory Medicine, Ningxia Medical University, Yinchuan, China

**Keywords:** *Klebsiella pneumoniae*, hypervirulence, co-producing, KPC-2, NDM-13

## Abstract

**Background:**

Carbapenem-resistant hypervirulent *Klebsiella pneumoniae* (CR-hvKp) co-producing KPC and NDM poses serious threats to public health. This study aimed to elucidate the molecular mechanisms underlying the resistance and virulence of a clinical CR-hvKP isolate coproducing KPC-2 and NDM-13 in China.

**Methods:**

The *K. pneumoniae* strain A51998714 was isolated from a 58-year-old male patient in China in June 2024. MALDI-TOF/MS was used to species identity. Antimicrobial susceptibility testing was determined using the VITEK-2 system. Whole-genome sequencing (WGS), plasmid conjugation assays, and plasmid stability testing were conducted to characterize resistance and virulence determinants. Virulence potential was assessed using serum-killing assays.

**Results:**

The CR-hvKp strain A51198714 exhibited resistance to nearly all tested antibiotics, including ceftazidime-avibactam and aztreonam. PCR and Sanger sequencing confirmed the co-occurrence of *bla*_KPC-2_ and *bla*_NDM-13_. WGS analysis revealed that strain A51198714, belonging to ST11-K64, possessed a single chromosome and five plasmids. Virulence-associated genes, localized on the IncHI1B plasmid pA51998714-VIR and the chromosome, were linked to enhanced colonization and infectivity. The serum-killing assay showed strain A51998714 was resistant to serum killing. The *bla*_KPC-2_, *bla*_CTX-M-65_, and *bla*_TEM-1_ genes were located on an IncFII plasmid pA51998714-KPC. The *bla*_NDM-13_ gene was located on plasmid pA51998714-NDM, which was classified as IncI1-I type. Conjugation assay and plasmid stability testing showed that plasmid pA51998714-NDM can be successfully transferred and maintained stably in the host.

**Conclusion:**

This study reports a clinical ST11-K64 CR-hvKP strain co-producing KPC-2 and NDM-13, which carried multiple resistance genes on two resistant plasmids, along with virulence factors enhancing its pathogenicity. These findings underscore the imperative for enhanced surveillance to mitigate the dissemination of such high-risk, multidrug-resistant, and hypervirulent strains.

## Introduction

*Klebsiella pneumoniae* (*K. pneumoniae*) is a Gram-negative bacillus of the *Enterobacteriaceae* family, ubiquitously distributed in natural environments and commonly colonizing the respiratory and intestinal tracts of humans and animals. It is recognized as an opportunistic pathogen and can cause a wide range of infections in humans (Li et al., 2025). Based on pathogenicity, *K. pneumoniae* can be categorized into two pathotypes: classical *K. pneumoniae* (cKp) and hypervirulent *K. pneumoniae* (hvKp) (Han et al., 2024). CKp is commonly associated with healthcare-associated infections and is known for its acquisition of various antimicrobial resistance genes. Carbapenem-resistant *Klebsiella pneumoniae* (CRKP) infections have been recognized as a critical global public health threat due to their association with elevated mortality and morbidity rates (Budia-Silva et al., 2024). The primary mechanism of carbapenem resistance is mediated through carbapenemase production. In China, *Klebsiella pneumoniae* carbapenemase (KPC) is the predominant carbapenemase, followed by New Delhi metallo-β-lactamase (NDM) in CRKP strains (Zuo et al., 2021). The hydrolytic activity of KPC can be effectively inhibited by new β-lactamase inhibitors, such as avibactam (AVI), vaborbactam, and relebactam. NDM enzymes belong to class B metallo-beta-lactamases (MbLs) and are not inactivated by currently commercialised β-lactamase inhibitors. In recent years, KPC and NDM-co-producing CRKP have been reported in most countries, especially in China, resulting limited treatment options for CRKP infection. In contrast to cKp, hvKp typically carries virulence plasmids (such as pLVPK-like), exhibiting a hypermucoviscous phenotype (hypervirulent phenotype), and hvKp is more virulent than cKP and sensitive to antimicrobials. HvKp was initially associated with community acquired invasive infections in healthy individuals, including pyogenic liver abscess, endophthalmitis, and metastatic infection (Bolourchi et al., 2022). It is generally believed that virulence plasmids of hvKp and carbapenem-resistant plasmids rarely stably coexist in the same strain. In recent years, *K. pneumoniae* strains have been identified integrating both carbapenem resistance and hypervirulence phenotypes, named carbapenem-resistant hypervirulent *K. pneumoniae* (CR-hvKp)(Zhou et al., 2023). Only a few sporadic reports of strains co-producing NDM and KPC are available to date, and most of these CR-hvKp strains are NDM-1-KPC-2-CR-hvKp strains or NDM-5-KPC-2-CR-hvKp strains. In this study, we isolated a clinical strain of ST11-K64 CR-hvKp co-producing KPC-2 and NDM-13. We present the antibiotic-resistant and hypervirulent phenotypes in detail, and further gain insight into the virulence and drug resistance mechanisms involved using whole-genome sequencing (WGS).

## Materials and methods

### Bacterial strain and antimicrobial susceptibility testing

Carbapenem-resistant *K. pneumoniae* A51998714 was isolated from a sputum sample from a 58-year-old male patient who was admitted to a hospital in China in June 2024. The isolate was identified by matrix-assisted laser desorption/ionization time-of-flight mass spectrometry (MALDI-TOF/MS) (bioMérieux, France). Antimicrobial susceptibility testing was conducted using the VITEK-2 system (bioMérieux, France) in accordance with the Clinical and Laboratory Standards Institute (CLSI) guidelines. The minimum inhibitory concentrations (MICs) of tigecycline were interpreted in accordance with the US Food and Drug Administration standards. The disk diffusion method was used to assess the synergistic activity of ceftazidime-avibactam and aztreonam.

### String test

The string test was performed as described previously (Hagiya et al., 2014). Briefly, the isolate was grown overnight at 37 °C on blood agar. A single colony was lifted with a loop to evaluate the formation of a viscous string between the loop and the colony. A positive string test was defined as a string length ≥ 5 mm.

### Phenotypic detection of carbapenemase production and detection of carbapenem resistance genes

Phenotypic detection assays were performed using combined-disc tests of imipenem alone and with phenylboronic acid (PBA) or EDTA or both PBA and EDTA, allowing discrimination between metal-β-lactamases (Class B carbapenemases, e.g., NDM) and KPC (Class A carbapenemases). PCR was performed for the detection of carbapenemase-encoding genes (*bla*_KPC_, *bla*_NDM_, *bla*_OXA-48_, and *bla*_IMP_) and positive amplifications were subjected to Sanger sequencing (Sangan Company, Shanxi, China). Sequences were analyzed using the BLAST program at the National Centre for Biotechnology Information.^1^ The primers used in this study are listed in Supplementary Table 1.

### Whole-genome sequencing (WGS) and bioinformatics analysis

Genomic DNA sequencing was conducted by Novogene Co., Ltd. (Beijing, China) using both Illumina HiSeq and PacBio platforms. The low-quality reads were filtered by the SMRT Link v8.0, and the filtered reads were assembled using the software Canu (https://github.com/marbl/canu/, version:2.0). Open-reading frames (ORFs) and pseudogenes were predicted using GeneMarkS (Version 4.17).^2^ Annotation of resistance genes, virulence factor genes, mobile elements, and other features was carried out using online databases including *CARD* (Alcock et al., 2020), *ResFinder* (Bortolaia et al., 2020), *VirulenceFinder* (Joensen et al., 2014; Malberg Tetzschner et al., 2020; Camacho et al., 2009), *VFDB* (Lihong Chen et al., 2005), *PlasmidFinder* (Carattoli et al., 2014), *ISfinder* (Siguier et al., 2006), and *INTEGRALL* (Moura et al., 2009). *Kleborate* was used to predict the MLST sequence type and serotype (Lam et al., 2021). Circular maps of plasmids were generated using the *BLAST Ring Image Generator (BRIG)* tool (Alikhan et al., 2011). The genetic environment was visualized by *EasyFig* software (Sullivan et al., 2011).

### Conjugation assays

The transfer ability of *bla*_KPC-2_ and *bla*_NDM-13_ harboring plasmids was assessed using *K. pneumoniae* A51998714 strain as the representative donor and *Escherichia coli* C600 as the recipient strain. Both the donors and C600 were grown to the exponential stage (the optical density at 600 nm reaches ~0.5) and then mixed at a donor/recipient ratio of 1:1. After incubation at 37 °C for 24 h, transconjugants were selected on MacConkey agar plates containing rifampicin 100 mg/L and meropenem 4 mg/L. The transconjugants were confirmed by PCR and Sanger sequencing. The antimicrobial susceptibility test of the transconjugants was confirmed by the VITEK-2 system.

### Plasmid stability assay

Plasmid stability testing was performed by the serial passage method for 10 consecutive days at 1:1000 dilutions without any antibiotic pressure (approximately 10 generations of growth per passage). The *E. coli* C600 transconjugants (C600/pA51998714-NDM) and *E. coli* C600 were propagated in antibiotic-free LB medium at 37 °C with 220 rpm shaking for 10 days. PCR detected the *bla*_NDM_ gene to determine its stability in an *E. coli* background.

### Growth rate determination

To investigate the fitness costs of the transconjugants (C600/pA51998714-NDM) in the absence of antibiotics, growth rate determination was performed as previously described (Zhu et al., 2023). Briefly, strains were grown overnight in 3 mL of LB with shaking (200 rpm) at 37 °C, and were then diluted to an OD600 of 0.25. Next, 2 μL of the solution was added to 200 μL LB in a 96-well plate in triplicate. Culture densities were determined every 10 min by measuring the OD600 for 12 h with shaking (200 rpm) at 37 °C. Relative growth rates were estimated using GraphPad Prism version 8 with a two-way analysis of variance (ANOVA), and a *p*-value < 0.05 was considered to be statistically significant.

### Serum-killing assay

*In vitro* virulence was evaluated using a serum-killing assay (Liu et al., 2024). Briefly, 25 μL of bacterial suspension (at a concentration of 1 × 10^6^ CFU/ mL) was added to 75 μL of mixed serum from healthy humans for co-culture in a microtiter plate. After 0, 1, 2, and 3 h, the plates were inoculated with the mixture, and the number of viable bacteria was determined. The test was independently performed at least three times, and the values of log10 CFU per milliliter were characterized as the result of serum resistance. Unpaired two-sided Student’s t-test was performed for the strains, and data were presented as means ± standard deviation (SD). *K. pneumoniae* strain ATCC 700603 was selected as a negative control, and the hypervirulent *K. pneumoniae* strain NTUH-K2044 was employed as a positive control strain.

## Results

### Clinical information and strain features of the CR-hvKP strain A51998714

**Figure 1:**
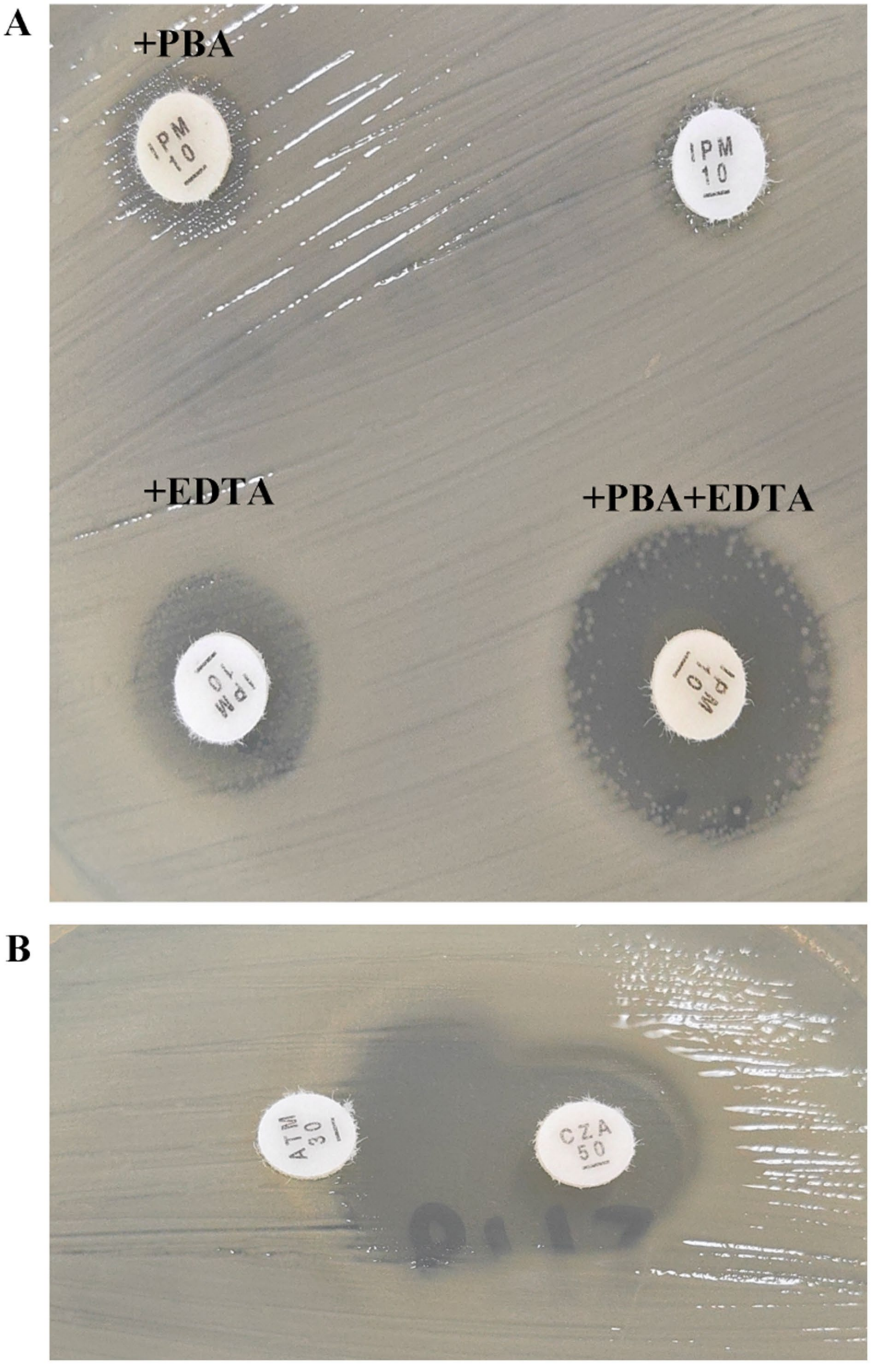
Identification of the carbapenemase production with combined-disc test and disk diffusion synergy test of CZA and ATM. **(A)** Combined discs consisting of imipenem alone, imipenem with PBA or EDTA respectively, or imipenem with PBA plus EDTA. Isolate showing positive combined-disc tests with enhanced zone of inhibition around both PBA and EDTA as compared to imipenem. **(B)** Disk diffusion synergy test of CZA and ATM. The results showed a synergistic effect between CZA and ATM. PBA: phenylboronic acid, EDTA: ethylenediaminetetraacetic acid, IMP: imipenem; CZA: ceftazidime-avibactam, ATM: aztreonam.

**Figure 2:**
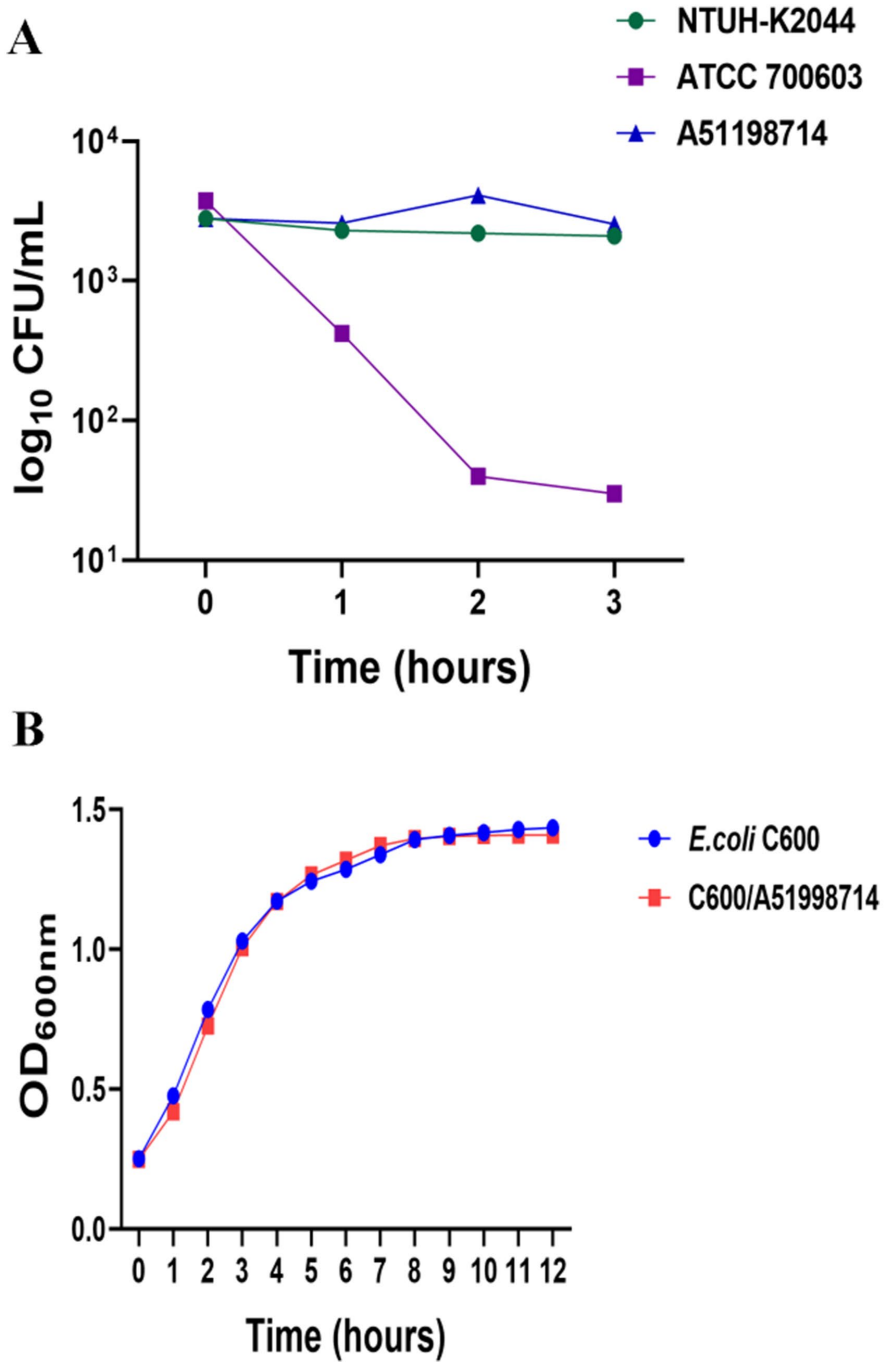
Serum-killing assay of strain A51998714 and growth curves of transconjugants. **(A)** Serum killing assay of strain A51998714. Survival of each strain was assessed by enumerating viable counts at 0, 1, 2, and 3 h of incubation in the pooled human sera at 37 °C. **(B)** Optical densities of transconjugant C600/A51998714 and *E. coli* C600 cultured in LB without antibiotic. The result showed no significant difference between transconjugant and *E. coli* C600 cu (*p* > 0.05).

The *K. pneumoniae* strain A51998714 was isolated from a 58-year-old male patient. The patient experienced intermittent chills and fever for over 1 month after pancreatic body and tail surgery 2 months ago, with symptoms worsening in the past 10 days. The patient’s condition worsened after treatment at a local hospital and was transferred to the general hospital of Ningxia Medical University in Jun-232024. The patient was diagnosed with severe pneumonia after admission. The strain *K. pneumoniae* A51998714 was isolated from sputum, and was resistant to nearly all the tested antibiotics, including imipenem, meropenem, cefepime, ceftazidime, aztreonam, amoxicillin-clavulanate, ceftazidime-avibactam, amikacin, tobramycin, levofloxacin, ciprofloxacin, but excluding tigecycline. The antimicrobial susceptibility profiles of *K. pneumoniae* A51998714 are presented in Table 1. The combined-disc test results indicated that the strain produces both serine carbapenemase and metallo-β-lactamase simultaneously (Figure 1A). The string test of A51998714 was negative, as the mucoid string length was < 5 mm. However, serum-killing assay indicated that the strain A51998714 was resistant to serum killing (Figure 2A). PCR and Sanger sequencing results showed the strain co-carrying *bla*_KPC-2_ and *bla*_NDM-13_ genes. The double disk synergy test showed a positive double disk synergy test between ceftazidime-avibactam and aztreonam (Figure 1B). The patient improved and was discharged after treatment with ceftazidime-avibactam in combination with aztreonam.

**Table 1:** Antimicrobial susceptibility of the clinical A51998714 srain and its conjugant.

Antimicrobial class	Antimicrobial agents	MIC(μg/mL)		C600
		A51998714	C600/pA51998714-NDM	
Cephalosporins	Cefepime	≥32 R	≥32 R	≤0.12 S
	Ceftazidime	≥64 R	≥64 R	0.5 S
	Ceftriaxone	≥64 R	≥64 R	≤1 S
β-lactam combination agents	Ticarcillin-clavulanate	≥128 R	≥128 R	≤8 S
	Ampicillin-sulbactam	≥32 R	≥128 R	4 S
	Piperacllin-tazobactam	≥128 R	≥128 R	≤4 S
Monobactams	Aztreonam	≥64 R	≤1 S	≤1 S
Carbapenems	Imipenem	≥16 R	≥16 R	≤0.25 S
	Meropenem	≥16 R	≥16 R	≤0.25 S
Aminoglycosides	Amikacin	≥64 R	≤2 S	≤2 S
	Gentamicin	≥16 R	≤1 S	≤1 S
	Tobramycin	≥16 R	≤1 S	≤1 S
Fluoroquinolones	Levofloxacin	≥8 R	0.5 S	0.5 S
	Ciprofloxacin	≥4 R	≤0.25 S	≤0.25 S
Folate pathway antagonists	Trimethoprim/sulfamethoxazole	≥320 R	≤20 S	≤20 S

### Sequence characteristics of the A51998714 genome

The complete genome sequence of the isolate was obtained, which consists of a 5,548,887-bp chromosome and five plasmids namely pA51998714-VIR (224,028 bp), pA51998714-KPC (126,326 bp), pA51998714-NDM (87,505 bp), pA51998714-1 (87,505 bp), and pA51998714-4 (84,883 bp) (Table 2). Plasmid pA51998714-1is a ColRNAI plasmid with no virulence genes and antibiotic resistance genes (ARGs). Plasmid pA51998714-4 is an unknown type plasmid carrying five antibiotic resistance genes, including *bla*_LAP-2_, *qnrS1*, *tet(A)*, *dfrA14*, and *sul2*. MLST analysis showed that A51998714 belongs to sequence type 11 and capsular serotype K64 (ST11-KL64). Prediction of ARGs showed that *K. pneumoniae* strain A51998714 carries multiple antibiotic resistance genes (ARGs) that confer resistance to beta-lactams (*bla*_NDM-13_, *bla*_KPC-2_, *bla*_LAP-2_, *bla*_SHV-11_, *bla*_TEM-1B_ and *bla*_CTX-M-65_), aminoglycosides (*aadA2b* and *rmtB*), sulfonamides (*sul1* and *sul2*), quinolones (*qnrS1*), trimethoprim (*dfrA14*), fosfomycin (*fosA6*) and tetracycline [*tet(A)*]. Most of the ARGs were located in plasmids pA51998714-KPC and pA51998714-NDM (Table 2). Using the *VFDB and VirulenceFinder* databases, genes in the A51998714 genome that may encode virulence factors were predicted. There are more than 80 virulence-related genes located on the chromosome and virulence plasmid pA51998714-VIR, including genes related to adherence, antiphagocytosis, efflux pump, iron uptake, regulation, secretion system and serum risistance (Table 3).

**Table 2:** Genomic characteristics of the clinical A51998714 srain.

Genetic material	Plasmid type	Size (bp)	GC content (%)	Antimicrobial resistance genes
Chromosome		5, 548, 887	57.31	*aadA2b*, *bla*_SHV-11_, *sul1*, *fosA6*
pA51998714-1	ColRNAI	11, 832	55.72	
pA51998714-KPC	IncFII	126, 326	53.11	*bla*_KPC-2_, *bla*_CTX-M-65_, *bla*_TEM-1B_, *rmtB*
pA51998714-VIR	IncHI1B	224, 028	50.09	
pA51998714-4	Unknowntype	84, 883	54.11	*bla*_LAP-2_, *qnrS1*, *tet(A)*, *dfrA14*, *sul2*
pA51998714-NDM	IncI1-I	87, 505	50.35	*bla*_NDM-13_

**Table 3:** Virulence related genes in the clinical A51998714 srain.

VF classes	Virulence factors	Related genes
Adherence	Type 3 fimbriae	*mrkA*, *mrkB*, *mrkC*, *mrkD*, *mrkF*, *mrkH*, *mrkI*, *mrkJ*
	Type I fimbriae	*fimA*, *fimB*, *fimC*, *fimD*, *fimE*, *fimF*, *fimG*, *fimH*, *fimI*, *fimK*
	Type IV pili biosynthesis(*Pseudomona*s)	*pilU*
	Type IV pili(*Yersinia*)	*pilQ*, *pilV*
Antiphagocytosis	Capsule	
Efflux pump	AcrAB	*acrA*, *acrB*
Iron uptake	Aerobactin	*iucA*, *iucB*, *iucC*, *iucD*, *iutA*
	Ent siderophore	*entA*, *entB*, *entC*, *entD*, *entE*, *entF*, *entS*, *fepA*, *fepB*, *fepC*, *fepD*, *fepG*, *fes*
	Salmochelin	*iroE*, *iroN*
	Yersiniabactin	*fyuA*, *irp1*, *irp2*, *ybtA*, *ybtE*, *ybtP*, *ybtQ*, *ybtS*, *ybtT*, *ybtU*, *ybtX*
Regulation	RcsAB	*rcsA*, *rcsB*
	RmpA	*rmpA*
Secretion system	T6SS-I	*clpV/tssH*, *dotU/tssL*, *hcp/tssD*, *icmF/tssM*, *impA/tssA*, *ompA*, *sciN/tssJ*, *tli1*, *tssF*, *tssG*, *vasE/tssK*, *vgrG/tssI*, *vipA/tssB*, *vipB/tssC*
	T6SS-II	*clpV*
	T6SS-III	*dotU*, *icmF*, *impA*, *impF*, *impG*, *impH*, *impJ*, *ompA*, *sciN*, *vgrG*
Serum resistance	LPS rfb locus	

### Sequence analysis of the virulence plasmid

**Figure 3:**
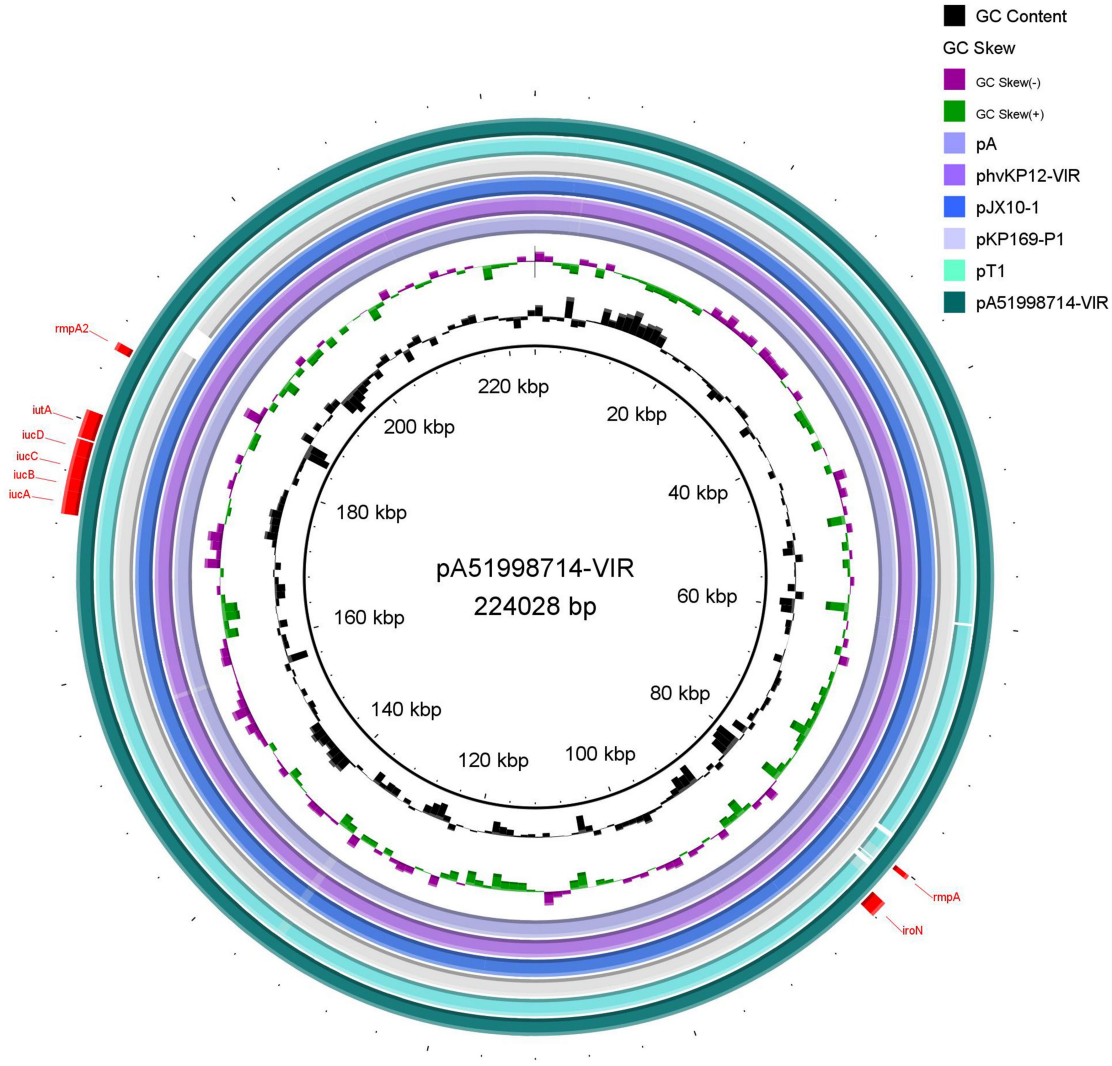
Genetic comparison of the virulence plasmid pA51998714-VIR. The plasmid pA51998714-VIR was used as the reference plasmid. Virulence genes are highlighted in red.

Virulence plasmid pA51998714-VIR was 224028bp, with an average GC content of 50.09%. A set of virulence genes in the IncHI1B plasmid pA51998714-VIR were associated with increased colonization and infection-producing capabilities (Figure 3): *iucABCD*-*iutA* and *iroN* for iron acquisition; *rmpA* and *rmpA2* for hypermucoviscous regulation; no antimicrobial resistance gene was identified. Comparative analysis with previously reported virulence plasmids showed that pA51998714-VIR had over 99% nucleotide identity and 98% query coverage with several plasmids: phvKP12-VIR (accession no. CP103316), pA (accession no. OM791346), pJX10-1 (accession no. CP064259), pT1_p1 (accession no. CP127154), and pKP169-P1 (accession no. CP078124).

### Characteristics of the IncFII plasmid carrying *bla*_KPC-2_ plasmid

**Figure 4:**
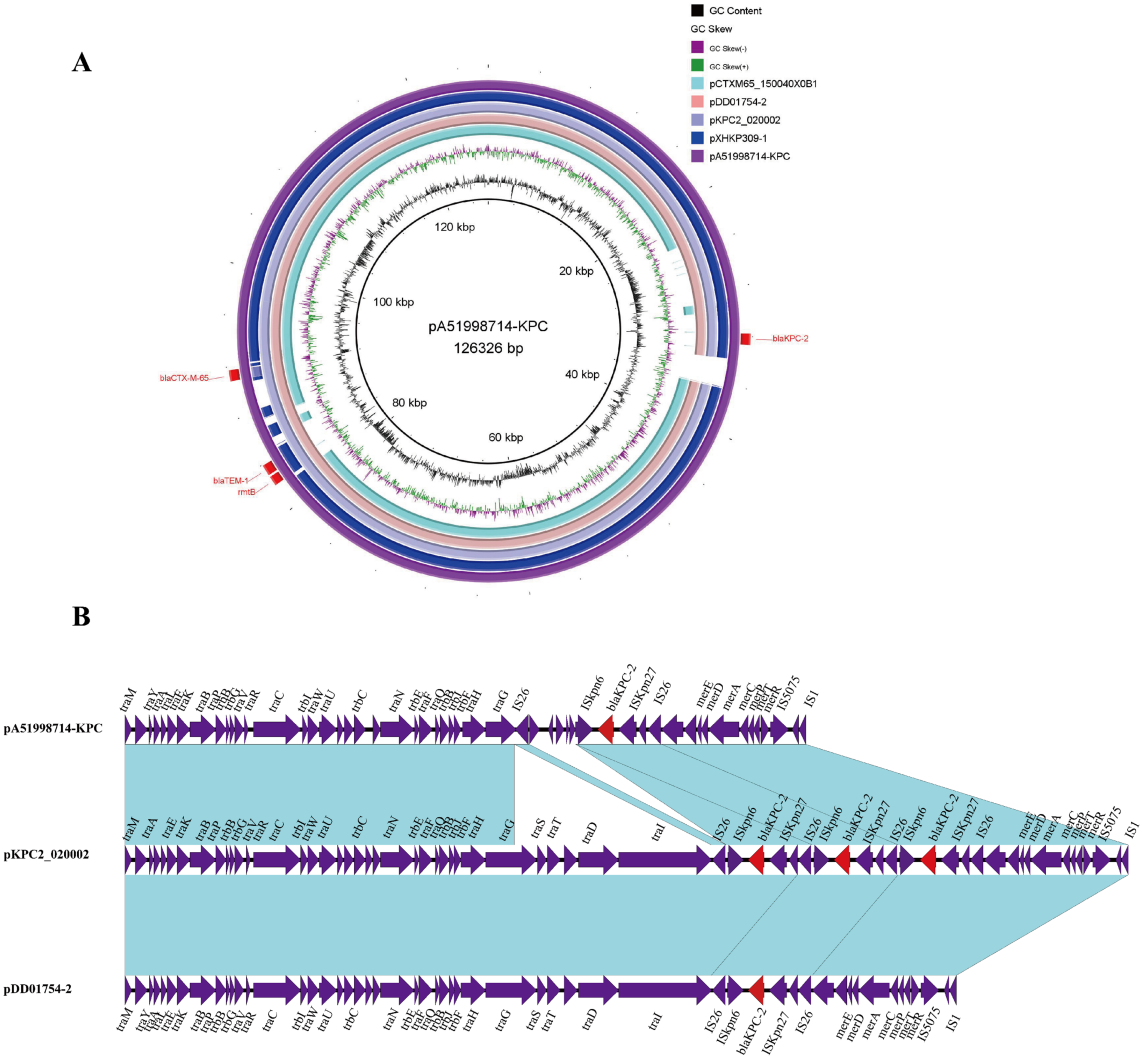
Comparative analysis between the pA51998714-KPC plasmid and other similar plasmids. **(A)** Circular comparison of plasmid pA51998714-KPC identified in this study with other similar plasmids and *bla*_KPC-2_-bearing plasmids in the NCBI database. The plasmid pA51998714-KPC was used as the reference plasmid. The red rectangles represent the resistance genes of the reference plasmid. Gaps show regions that are missing in the respective plasmids compared with the reference plasmid. **(B)** Schematic illustration comparing the structural features of plasmid pA51998714-KPC with sequences of plasmid pKPC2_020002 (NZ_CP028541.2) and pDD01754-2 (NZ_CP087647.1). Blue shading and squares indicate homologies between the corresponding genetic loci on each plasmid. Arrows indicate open reading frames, and red arrows indicate blaKPC-2 gene.

Plasmid pA51998714-KPC carries the carbapenem resistance gene *bla*_KPC-2_, which is 126,326 bp in size and has a GC content of 53.11%. It was identified as a typical IncFII plasmid, which is the most common type of plasmid carrying the *bla*_KPC-2_ gene. In addition to the *bla*_KPC-2_ gene, *bla*_CTX-M-65_, *bla*_TEM-1_, and *rmtB* genes were also identified on the same plasmid. BLASTn search results showed that pA51998714-KPC shared high similarity with many plasmids, including pDD01754-2 (97.61% query coverage and 99.94% identity, accession no. CP087647), pKPC2_020002 (97.61% query coverage and 99.94% identity, accession no. CP028541), pXHKP309-1 (93.69% query coverage and 99.93% identity, accession no. CP066901), plasmid unnamed2 (86.14% query coverage and 99.95% identity, accession no. CP107299), and pCTXM65_150040X0B1 (86.1% query coverage and 100% identity, accession no. CP104031) (Figure 4A). Further analysis revealed that *bla*_KPC-2_ gene in the pA51998714-KPC plasmid was not associated with the Tn*4401* transposon, which was a major transposon associated with the *bla*_KPC-2_ gene in *Enterobacteria*. The *bla*_KPC-2_ structure was IS*26-*ISKpn*6*-*bla*_KPC-2_-ISKpn*27*-IS*26*, which was similar to two previously sequenced plasmids (pDD01754-2 and pKPC2_020002) in two ST11 *K. pneumoniae* strains DD01754 and WCHKP2 from China (Feng et al., 2019; He et al., 2024). The primary difference among the three plasmids is that there are three copies of the IS*26*–ISKpn*27*–*bla*_KPC-2_–ISKpn*6*–IS*26* unit present in tandem on plasmid pKPC2_020002. On plasmid pA51998714-KPC, *IS*26 and *ISKpn*6 were truncated by ~1.2 kb sequence (Figure 4B).

### Characteristics of the IncI1 plasmid carrying *bla*_NDM-13_

**Figure 5:**
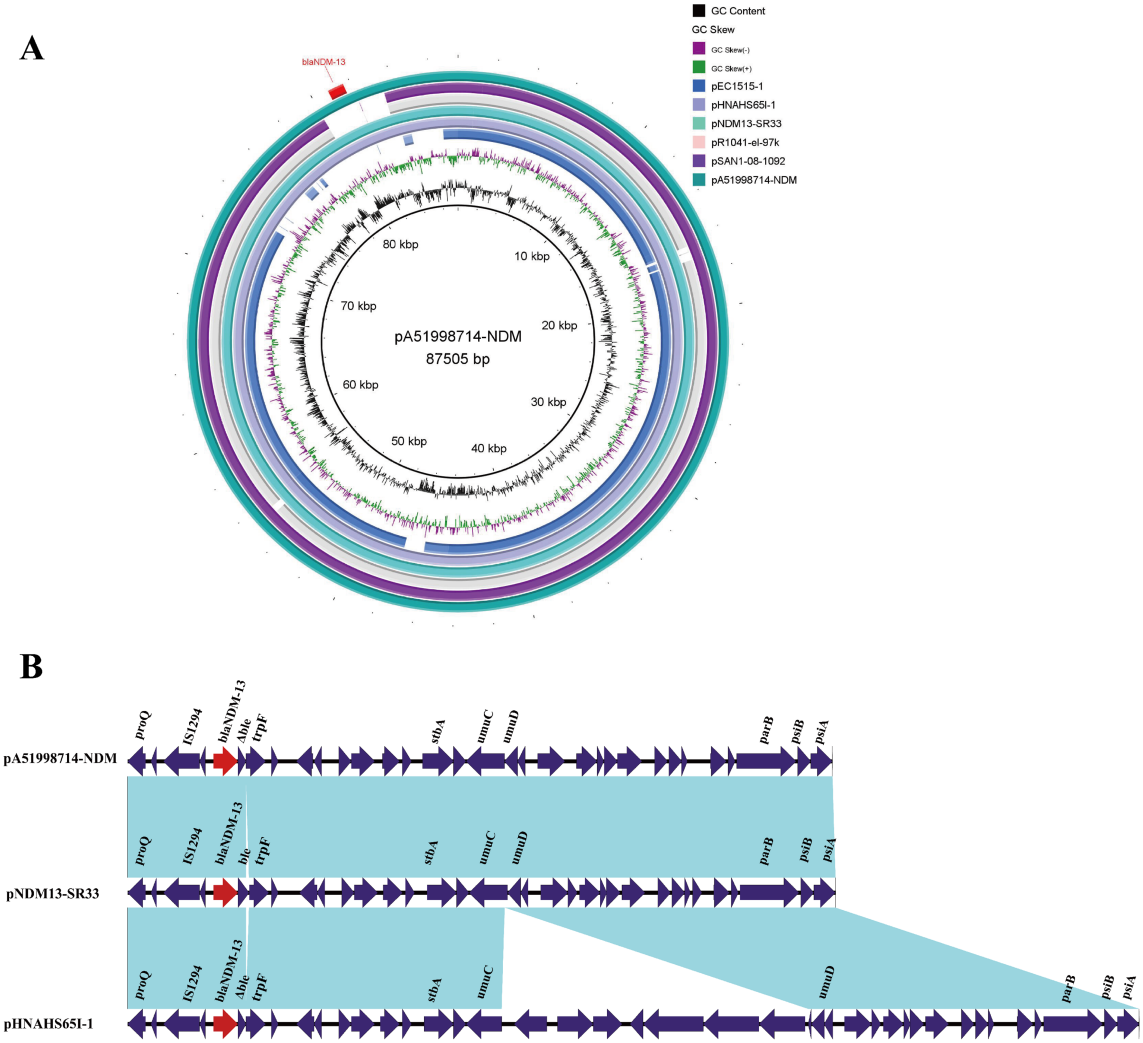
Comparative analysis between the pA51998714-NDM plasmid and other similar plasmids. **(A)** Circular comparison of plasmid pA51998714-NDM identified in this study with other similar plasmids and *bla*_NDM_-bearing plasmids in the NCBI database. The plasmid pA51998714-NDM was used as the reference plasmid. The red rectangles represent the resistance genes of the reference plasmid. Gaps show regions that are missing in the respective plasmids compared with the reference plasmid. **(B)** Schematic illustration comparing the structural features of plasmid pA51998714-NDM with sequences of plasmid pNDM13-SR33 (NZ_CP092912.1) and pHNAHS65I-1 (NZ_MN219406.1). Blue shading indicate homologies between the corresponding genetic loci on each plasmid. Arrows indicate open reading frames, and red arrows indicate *bla*_NDM-13_ gene.

Plasmid pA51998714-NDM is an 88,156-bp IncI1 plasmid with an overall GC content of 50.35% (Figure 5A). pA51998714-NDM carries the carbapenem resistance gene *bla*_NDM-13_, which is identified as a typical IncI1 plasmid and contains regions involved in plasmid stability, replication, and conjugative transfer. BLAST comparison indicated that pA51998714-NDM showed an overall nucleotide identity (99.73–99.99%) and query coverage (83.82–99.95%) similar to several plasmids, such as pR1041-el-97 k (accession no. OR095751), pNDM13-SR33 (accession no. CP092912), pHNAHS65I-1 (accession no. MN219406), pEC1515-1 (accession no. CP021845) and pSAN1-08-1092 (accession no. CP019996) that have been reported in different countries. Of note, plasmids pNDM13-SR33 and pHNAHS65I-1 carried the *bla*_NDM-13_ gene and belonged to the IncI1 plasmid, too. The plasmid pA51998714-NDM shared 99% coverage and 100% identity with plasmid pNDM13-SR33 of a *Salmonella Rissen* ST469 clinical isolate from a fecal sample of an old patient in China. pA51998714-NDM shared 99% coverage and 100% identity with plasmid pHNAHS65I (accession no. MN219406) carried by an *E. coli* strain discovered in 2020 (Figure 5A). In addition, bioinformatics analysis showed that the *bla*_NDM-13_ gene of pA51998714-NDM was organized as ΔISAba*125*-IS*1294*-ΔISAba*125*-*bla*_NDM-13_-*ble*_MBL_-trpF. The same genetic structure was observed in plasmids pNDM13-SR33 and pHNAHS65I, as shown in Figure 5B.

### The biological features of plasmids pA51998714-NDM

The plasmid pA51998714-NDM can be successfully transferred to the *E. coli* C600 recipient strain. AST results showed that the *E. coli* transconjugant C600/pA51998714-NDM carrying pA51998714-NDM exhibits increased MICs towards carbapenems (imipenem, meropenem), cephalosporins (cefepime, ceftazidime), and β-lactam/β-lactamase inhibitors (amoxicillin-clavulanate, ceftazidime-avibactam) (Table 1). However, transconjugants carrying pA51998714-KPC were not obtained, despite repeated attempts. To assess the stability of pA51998714-NDM, we performed passage experiments with the transconjugant C600/pA51998714-NDM in the absence of antibiotics for 10 days. The stability of pA51998714-NDM reached 100% in the absence of antibiotics after 10 days of passing between generations, indicating that the plasmid can be stably inherited in *E. coli* C600. To further evaluate the effect of the IncI1 plasmid carrying *bla*_NDM-13_ on growth, we compared the growth of the transconjugants C600/A51998714 and *E. coli* C600 in the absence of antibiotics. The results showed no significant difference (*p* > 0.05) between C600/pA51998714-NDM and *E. coli* C600 (Figure 2B).

## Discussion

Carbapenem-resistant hypervirulent *Klebsiella pneumoniae* (CR-hvKp) poses a serious threat to public health. However, the increasing reports of CR-hvKp co-harboring *bla*_KPC_ and *bla*_NDM_ have posed greater challenges to clinical treatment. The first documented case of CR-hvKp simultaneously producing KPC and NDM was reported in China, followed by sporadic reports in other regions. Commonly, the predominant KPC subtypes reported are KPC-2 and KPC-3, while the main NDM subtypes are NDM-1 and NDM-5 (Zhou et al., 2024; Ahmed et al., 2021). Lu et al. first reported *K. pneumoniae* strains co-producing KPC-2 and NDM-13, which accounted for a significant proportion in their study, highlighting the need for enhanced surveillance of KPC-2- and NDM-13-positive strains in China (Zhang et al., 2025). In this study, we isolated a clinical CR-hvKP strain co-producing KPC-2 and NDM-13. Antimicrobial susceptibility testing revealed near-pan-drug resistance, including resistance to ceftazidime-avibactam and aztreonam. However, disk diffusion synergy testing demonstrated a synergistic effect between ceftazidime-avibactam and aztreonam. The patient was successfully treated with ceftazidime-avibactam combined aztreonam, and discharged in improved condition. Consistent with the previous findings, strains co-producing KPC and NDM may be treated with ceftazidime-avibactam in combination with aztreonam, indicating that dual-carbapenemase producers could benefit from this therapeutic approach (Tamma et al., 2023; Gupta et al., 2024). Liu et al. (2019) isolated an ST86 hvKP strain that co-produces NDM-1 and KPC-2 carbapenemases in Nanchang, China. Liu et.al (Hao et al., 2022) reported a hypervirulent ST464 *K. pneumoniae* strain cocarrying *bla*_NDM − 1_ and *bla*_KPC − 2_ in Southwestern China. Meanwhile, the increasing prevalence of hypervirulent carbapenem-resistant *K. pneumoniae* has been documented in numerous countries (Chen et al., 2020; Kochan et al., 2022; Mohammed et al., 2024; Lorenzin et al., 2022). These findings emphasize the need for monitoring of the CR-hvKP.

The string test is a widely used clinical method for identifying hypervirulent strains of *K. pneumoniae*, but the strain A51998714 in this study was string-test-negative and did not exhibit a hypermucoviscous phenotype. Cai et al. reported a clinical hvCRKP was non-hypermucoviscous and lacked *magA* and *rmpA* genes and pLVPK plasmid but exhibited high virulence (Cai et al., 2022). The whole-genome sequencing confirmed the presence of multiple virulence genes located on both chromosomes and plasmids in strain A51998714, including rmpA and iucA related to hypermucoviscous phenotype (Jia et al., 2024). Recent studies have increasingly shown that the string test does not encompass all hypervirulent strains. Some CRKP isolates, despite being string-test-negative, still carry various virulence genes and demonstrate high pathogenicity. This phenomenon challenges the conventional definition of hvKP and underscores the need for more comprehensive virulence assessment methods (Cai et al., 2022; Russo et al., 2024).

The coexistence of virulence and resistance plasmids in *K. pneumoniae* poses a significant challenge in clinical settings, as it enhances both pathogenicity and antimicrobial resistance. In this study, we characterized four plasmids in the CR-hvKp strain A51998714, including one virulence plasmid (pA51998714-VIR) and two resistance plasmids (pA51998714-KPC and pA51998714-NDM), highlighting their genetic features and potential contributions to bacterial fitness and resistance.

The IncHI1B virulence plasmid pA51998714-VIR harbored key virulence determinants, including *iucABCD*-*iutA*, *iroN*, and *rmpA/rmpA2*, which are known to enhance bacterial survival and pathogenicity in host environments (Russo, 2019). The absence of resistance genes suggests that this plasmid is specialized for virulence, potentially facilitating severe infections such as liver abscesses or bloodstream infections. The high nucleotide identity (>99%) with previously reported virulence plasmids (e.g., phvKP12-VIR, pA, pJX10-1) indicates a conserved genetic backbone among hvKP strains, possibly due to horizontal gene transfer (HGT) and clonal dissemination. This finding underscores the risk of CR-hvKP emergence, which could lead to difficult-to-treat infections.

*K. pneumoniae* carbapenemase-2 (KPC-2) is the most common carbapenemase resulting in CRKP, which is encoded by *bla*_KPC-2_ gene (Song et al., 2024). The *bla*_KPC-2_ gene is frequently carried by IncFII-type plasmids (Jiang et al., 2025). Our results showed that the *bla*_KPC-2_ gene in CR-hvKP strain A51998714 is located on an IncFII plasmid pA51998714-KPC. Meanwhile, the plasmid pA51998714-KPC carried other resistance genes, including *bla*_CTX-M-65_, *bla*_TEM-1_, and *rmtB*, conferring resistance to extended-spectrum *β*-lactams, and aminoglycosides. The high similarity (>99% identity) with plasmids such as pDD01754-2 and pKPC2_020002 (from ST11 *K. pneumoniae* strains in China) suggests clonal expansion of this plasmid among high-risk CRKP clones. Notably, the gene *bla*_KPC-2_ in pA51998714-KPC was flanked by IS*26*-ISKpn*6*-*bla*_KPC-2_-ISKpn*27*-IS*26*, a non-*Tn*4401 elements previously reported in Chinese (He et al., 2024). The absence of Tn*4401*, the classical *bla*_KPC-2_ transposon, indicates alternative mobilization mechanisms, possibly mediated by IS*26*-driven recombination. The truncation of IS*26* and ISKpn*6* in pA51998714-KPC suggests ongoing genetic rearrangements, which may influence plasmid stability and resistance gene dissemination.

New Delhi metallo-b-lactamase-13 (NDM-13) is an NDM variant that was first identified in 2015 (Shrestha et al., 2015). To date, only sporadic studies investigate *bla*_NDM-13_-carrying strains, including *E. coli* strains and one clinical *Salmonella Rissen* isolate (Zhang et al., 2023; Ju et al., 2023; Kim et al., 2019; Huang et al., 2022). It is reported that *bla*_NDM-13_ genes are primarily located on conjugative plasmids, including two IncI1 plasmids pHNAHS65I-1 (accession no. MN219406) and pNDM13-SR33 (accession no. CP092912), an IncX3 plasmid pNDM13-DC33 (accession no. KX094555), and an IncFIB plasmid pSECR18-0956 (accession no. MK157018). In this study, the plasmid pA51998714-NDM carried *bla*_NDM-13_ identified as a typical IncI1 plasmid. Of note, pA51998714-NDM shared 99.95% coverage and 99.98% identity with the plasmid pNDM13-SR33 of *Salmonella Rissen* discovered in 2022, and 99.95% coverage and 99.99% identity with the plasmid pHNAHS65I-1 of *E. coli* discovered in 2020. Moreover, the genetic context of *bla*_NDM-13_ in A51998714 was ΔISAba*125*-*bla*_NDM-13_-*ble*_MBL_-trpF, a structure identical to plasmids pNDM13-SR33 and pHNAHS65I-1 (Ju et al., 2023; Huang et al., 2022). These findings suggest interspecies transmission of *bla*_NDM-13_ within *Enterobacteriaceae*, facilitated by the highly conjugative IncI1 backbone. The persistence of such plasmids in clinical isolates raises concerns about their role in carbapenem resistance spread, particularly in regions where NDM producers are endemic. Among these NDM variant genes, *bla*_NDM-1_ and *bla*_NDM-5_ are the two predominant NDM carbapenemase genes (Shen et al., 2022). The vast majority of *bla*_NDM_ genes are located on plasmids, which can spread among several hosts, facilitating their dissemination. IncFI was the most dominant type, followed by IncX3 and IncA/C, and IncX3 is the dominant Inc. in China (Li et al., 2023). However, our results may indicate that carriage of *bla*_NDM-13_ on a transmissible IncI1 plasmid may result in an increased risk of *bla*_NDM-13_ transmission.

## Conclusion

This study underscores the importance of plasmid surveillance in CRKP, as virulence and resistance plasmids can co-evolve and spread rapidly among bacterial populations. The genetic flexibility of these plasmids, mediated by mobile elements, facilitates their persistence and transmission across species. Future studies should investigate the clinical impact of such strains and explore strategies to limit their dissemination, such as enhanced infection control and antibiotic stewardship.

## Data Availability

The datasets presented in this study can be found in online repositories. The names of the repository/repositories and accession number(s) can be found at: https://www.ncbi.nlm.nih.gov/genbank/, SAMN50543836. The complete sequences of chromosome and plasmids from A51998714 were submitted to GenBank under BioProject PRJNA1303965.

## Footnotes

1
http://blast.ncbi.nlm.nih.gov/Blast.cgi

2
http://topaz.gatech.edu/GeneMark/
